# Isolation and Antibiotic Susceptibility Testing of *Haemophilus influenzae* from Nasopharynx of Children under Five Years Attending Maternal and Child Health Clinic in Mbarara Regional Referral Hospital

**DOI:** 10.1155/2019/6542919

**Published:** 2019-03-03

**Authors:** Daniel Omoding, Joel Bazira

**Affiliations:** ^1^Mbarara University of Science and Technology, Mbarara, Uganda; ^2^Infectious Diseases Research Collaborations, Mbarara, Uganda

## Abstract

*Background. H. influenzae* remains an organism of a major public health challenge worldwide despite the availability of the Hib vaccine, particularly among children under 5 years. Information on the current carriage status and antibiotic susceptibility is key on proper health-care provision. Therefore, we conducted a study to determine *H. influenzae* carriage rate and antibiotic susceptibility testing of the isolates among the children. *Methods*. This was a cross-sectional study conducted between January and May 2018, among clinically healthy children under five years attending Maternal and Child Health (MCH) Clinic in Mbarara Regional Referral Hospital (MRRH). We carried out standard microbiology methods to culture, isolate, and identify *H. influenzae*, and then, we tested for their susceptibility to commonly used antibiotics following the CLSI standards. *Results*. Of the 248 participants included in the study, 116 (46.77%) were females and 132 (53.23%) males and 78 (31.45%) were below the age of 3 months. Fifty one of the study participants had *H. influenzae* in their nasopharynx, which represents 20.56% carriage (95% CI 15.49 to 25.63). There was a general high susceptibility of the isolates to the antimicrobial agents commonly used. There was 100% susceptibility to ciprofloxacin and imipenem antibiotic agents, though 6 (11.76%) and 4 (7.84%) of the isolates showed resistance to chloramphenicol and ampicillin, respectively. *Conclusion*. The high burden presented by *H. influenzae* and the resultant impact on child health require much attention to prevention of infections associated with the organism. A well-funded molecular study focusing on typing the isolates would determine the impact of the vaccine, given the carriage rates are still high.

## 1. Background

*Haemophilus influenzae* is a Gram-negative pleomorphic, anaerobic pathogenic bacterium that can be either encapsulated or unencapsulated, referred to as nontypeable *H. influenzae* (NTHi) [1]. Six antigenically and biochemically distinct serotypes (a, b, c, d, e, and f) have been described [2], of which type b strains are notoriously associated with invasive disease. Normal individuals can carry *H. influenzae* in their naso- and oropharynx, a major factor inducing the development of natural immunity against the pathogen [3], though it is one of the main pathogens that cause community-acquired respiratory tract infections during childhood [4]. Asymptomatic carriers can still transmit to other individuals, with disease resulting if the pathogens descend the airway into the lower respiratory tract [1].

The rate of carriage of *H. influenzae* increases from infancy (about 20% in the first year of life) to early childhood (50% in children aged 5 to 6 years), and *H. influenzae* is recoverable from the upper airways of 20 to 80% of healthy children [5]. Studies done in China, Australia's Northern Territory, and Kenya among children under 5 years recorded 26.33% with 31% of the isolates producing beta-lactamase [6], 36.5% [7], and 26% [8], respectively.

*H. influenzae* can cause a variety of infections ranging from minor localized to very invasive systemic infections which include *H. influenzae* b (Hib) meningitis, cellulitis, epiglottitis, Hib pneumonia, Hib pericarditis, septic arthritis, and occult bacteremia and underlying medical conditions (especially with invasive Hib disease) such as pulmonary disease, immunodeficiency states (e.g., HIV infection), alcoholism, pregnancy, malignancy, and neonatal infections. NTHi is associated with various mucosal infections, causing common pediatric diseases, including otitis media (OM) [9, 10] and conjunctivitis and with exacerbations of chronic obstructive pulmonary disease (COPD) in adults [11]. Carriers of NTHi are healthy but occasionally develop localized acute respiratory tract infections such as otitis media, sinusitis, pneumonia, and conjunctivitis [12].

Prior to the introduction of type b *H. influenzae* vaccines, Hib was thought to be responsible for approximately three million serious illnesses and an estimated 386,000 deaths per year, with 95% of cases and 98% of deaths worldwide occurring among patients in developing countries [13]. Children between 4 and 18 months of age are the most vulnerable to infections caused by *H. influenzae*, being the second most common pathogen causing pneumonia in Chinese children and accounting for 30–50% of bacterial meningitis [14, 15].

A minimum of 3% of patients with Hib meningitis die even with prompt and adequate antibiotic treatment [16] and may lead to hearing loss in 3% to 6% of children in developed countries [17], which may reflect delay in accessing treatment, suboptimal case management, the influence of associated conditions (such as HIV infection or malnutrition), or other factors [18].

Pediatric vaccination against Hib has resulted in a dramatic decrease in the incidence rates of invasive Hib disease in all countries where the vaccine has been included in the national immunization programs [19], but there is a notable existence of infections caused by *H. influenzae*. Infections caused by NTHi notably reported worldwide, suggesting strain replacement [2, 20].

It was therefore necessary to determine the precise carriage rate of *H. influenzae* after introduction of Hib vaccine and determine antibiotic susceptibility of the isolates.

## 2. Methods

We conducted a cross-sectional study in Mbarara Regional Referral Hospital (MRRH), Mbarara, Uganda, from January to May 2018. Nasopharyngeal (NP) swabs were collected from clinically healthy children (with no history of respiratory illness or antibiotic use in the past 3 weeks) below five years, attending Maternal and Child Health (MCH) Clinic for immunization by convenience sampling. An NP swab was inserted into one nostril straight back (not upwards) and horizontally to the nasopharynx up to the measured distance on the swab handle and rotating the swab up to 5 times and holding in place for 5–10 seconds to collect sample material. The swab was removed and inserted into a tube containing 1.5 ml of brain heart infusion broth glycerol (BHI-glycerol) and sent to the Microbiology Laboratory for culture. The study was approved by the Faculty Research Ethics Committee (FREC) and Institutional Review Committee (IRC) of Mbarara University of Science and Technology, and the parents/caretakers provided written informed consent. The tube containing the NP swab sample was vortex mixed, and a drop inoculated on a chocolate agar plate containing bacitracin (20,000 U/L of media) and incubated at 37°C and carbon dioxide. Bacitracin inhibits growth of many other normal floras, allowing easy isolation of *H. influenzae*. All isolates were confirmed by standard laboratory methods on the basis of colony morphology, Gram's stain, oxidase test, and X- and V-factor requirement as *H. influenzae.* Haemolysis on 5% sheep blood was used to differentiate *H. influenzae* from *H. haemolyticus* (*H. influenzae* is nonhaemolytic while most strains of *H. haemolyticus* produce beta-haemolysis). 0.5 McFarland turbidity of each isolate was prepared in sterile peptone water, inoculated on chocolate agar plates, and tested against a select antibiotic discs commonly used in the lab. Their zones of inhibitions recorded and interpreted as “Sensitive (*S*),” “Intermediate (*I*),” or “Resistant (*R*),” according to CLSI guidelines [21]. Data were analyzed using Microsoft Excel and STATA 12 programs, and results were presented in tables, charts, and graphs.

## 3. Results

**Figure 1:**
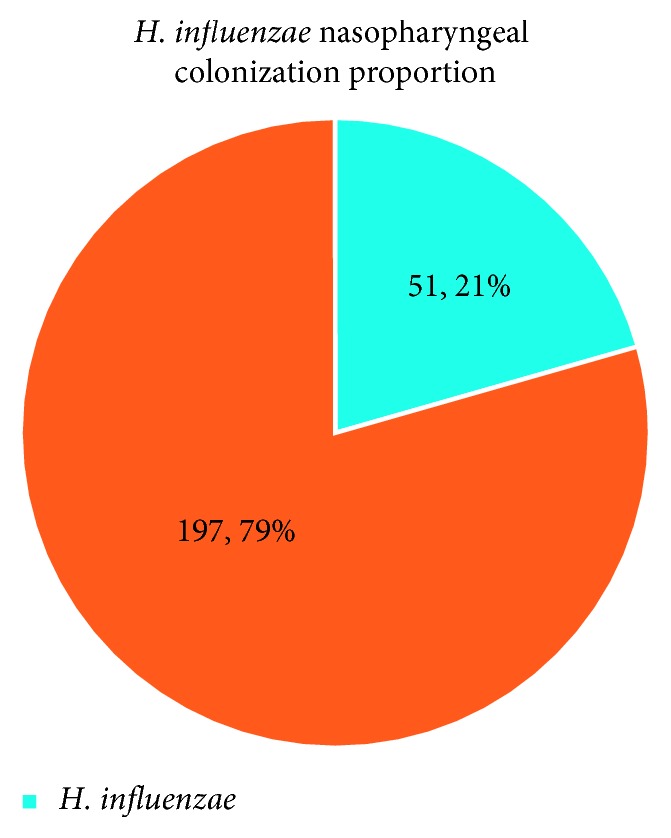
The proportion of participants with *H. influenzae*.

**Figure 2:**
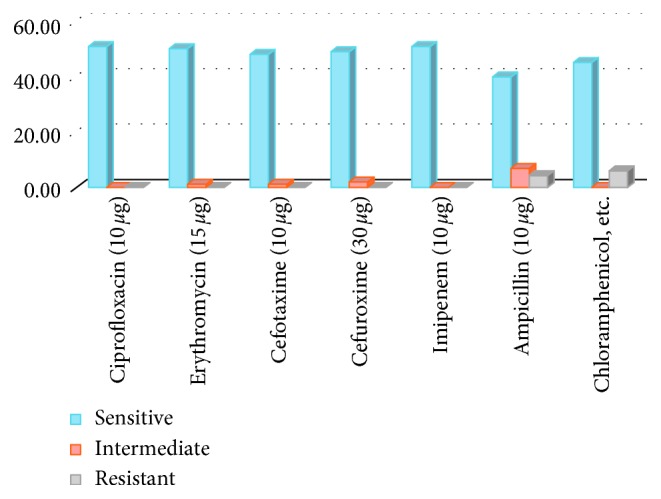
Drug susceptibility test of the *H. influenzae* isolates.

248 children aged 1 to 60 months were recruited in the study in a period of 3 months from 23rd Jan to 13th May 2018. Fifty one out of 248 children carried *H. influenzae* in their nasopharynx, and there was a high susceptibility to the commonly used antibiotics. Seventy eight (31.45%) of the children who participated in the study were below the age of 3 months, and there was no significant difference between the number of males and females (*p*=0.556) (Table 1). Fifty one of the children had nasopharyngeal colonization with *H. influenzae*, which represented a prevalence of 20.56, 95% CI (15.49 to 25.63) (Figure 1). There was no statistical difference in carriage between males and females (*p*=0.718). The carriage rates were highest among children aged 6 to 8 months, followed by the children aged 9 to 11 months, and reduced with age. The least carriage rates were recorded among children below 3 months followed by those aged 3 to 5 months. However, the difference in carriage among the different age categories was not statistically significant (*p*=0.197) (Table 2). There was a generally high sensitivity of the isolates to the antimicrobial agents commonly used. There was 100% sensitivity to ciprofloxacin and imipenem antibiotic agents. However, 6 (11.76%) and 4 (7.84%) of the isolates showed resistance to chloramphenicol and ampicillin, respectively. Also, 1 (1.96%), 3 (5.88%), 2 (3.92%), and 7 (13.73%) of the isolates were intermediate against erythromycin, cefotaxime, cefuroxime, and ampicillin, respectively. Three isolates were resistant to both chloramphenicol and ampicillin, 2 isolates were resistant to chloramphenicol but intermediate to ampicillin, 1 isolate was resistant to ampicillin but intermediate to erythromycin, cefotaxime, and cefuroxime, 1 isolate was resistant to chloramphenicol, 5 isolates were intermediate to ampicillin, and 1 isolate was intermediate to cefotaxime and cefuroxime (Figure 2).

## 4. Discussion

*H. influenzae* remains a microorganism of interest in developing countries with children under the age of 5 years at risk of suffering from diseases associated with the organism. From this study, a carriage rate of 21% was found, which is high and provides a ground for infections associated with the organism. The results obtained in this study agree with the studies done by Fontanals among the Catalan preschool population [5] and Zhu among children younger than 5 years of age in Beijing, China [6].

The carriage rate of 21% was however less than that obtained in a study done among Australia's Northern Territory (NT) Aboriginal population to determine respiratory causes responsible for deaths, which reported 36.5% [7] and a study done in Kilifi District, Kenya, which reported 26% nasopharyngeal carriage among children under five years [8].

This generally shows that despite introduction of Hib vaccine, the carriage rates of *H. influenzae* are still high though they are expected to drop, suggesting the possibility of strain replacement, where Hib which used to be the most prevalent is now being replaced by non-Hib *H. influenzae*. This could also explain persistent infections associated with *H. influenzae* despite introduction of the vaccine.

The carriage rates were lowest among the children below 3 months of age, followed by those aged 3 to 5 months, increased to the highest rates recorded among 6- to 8-month-aged children and reduced as the children grew older. This indicates existence of maternal antibodies among the children aged up to 5 months, which provide them with immunity against the organisms. These antibodies clear as the baby grows, before starting to produce its own antibodies, making the children aged 7 to 11 months the most vulnerable to the infection [14, 15]. It could also be that a majority of the under 6 months old have not had sufficient exposure to pick up the organisms from the environment. The fact that the difference in carriage among the different age groups was not statistically significant, suggests that there could be other factors, not looked at in this study, that determine the carriage status.

The isolates showed a generally high sensitivity to antibiotic agents, with 100% sensitivity to quinolones and carbapenems. The 21% failure of the isolates to be sensitivity to ampicillin indicates the possibility of beta-lactamase activity. The overall high sensitivity of the isolates to antibiotic agents indicates that there was no overuse of antibiotics by the population.

## 5. Conclusion

A nasopharyngeal carriage *H. influenzae* of 21% among children under 5 years attending Maternal and Child Health Clinic in MRRH is high, given the existence of Hib vaccine. There is therefore a need to investigate if Hib is still predominant or there has been replacement of strains by nontype b *H. influenzae*.

## Figures and Tables

**Table 1:** The distribution of participants by gender and age.

Age intervals (months)	Sex		Total
	Female	Male	
Below 3	39 (50.00%)	39 (50.00%)	78 (31.45%)
3 to 5	17 (38.64%)	27 (61.36%)	44 (17.74%)
6 to 8	10 (40.00%)	15 (60.00%)	25 (10.08%)
9 to 11	21 (51.22%)	20 (48.78%)	41 (16.53%)
12 to 18	16 (45.71%)	19 (54.29%)	35 (14.11%)
Above 18	13 (52.00%)	12 (48.00%)	25 (10.08%)
Total	116 (46.77%)	132 (53.23%)	248 (100.00%)

**Table 2:** Carriage in different gender and age groups.

Variable	No. included	Carriage
*Sex*		
Female	116	25 (21.55%)
Male	132	26 (19.70%)
*Age (months)*		
Below 3	78	11 (14.10%)
3 to 5	44	7 (15.91%)
6 to 8	25	9 (36.00%)
9 to 11	41	11 (26.83%)
12 to 18	35	8 (22.86%)
Above 18	25	5 (20.00%)

## Data Availability

Raw data of the study can be found at https://www.dropbox.com/s/avczcginyykmswn/Data-Hi%20Carriage.xlsx?dl=0.
